# Evaluation of the Cytocompatibility of Fluoride Varnish and Its Effect on Human Gingival Fibroblasts (hGFs): An In Vitro Study

**DOI:** 10.7759/cureus.41735

**Published:** 2023-07-11

**Authors:** V. Ranjith Akshay Seshadri, Nikitha S Varghese, Deepa Gurunathan

**Affiliations:** 1 Pediatric and Preventive Dentistry, Saveetha Dental College, Saveetha Institute of Medical and Technical Sciences, Chennai, IND

**Keywords:** apoptosis, concentrations, oral fibroblasts, cytotoxicity, fluoride varnish

## Abstract

Introduction

Untreated dental decay poses a significant oral health challenge, leading to pain, tooth loss, and infections. Fluoride varnishes are in prolonged contact with the tooth surface and this prevents dental decay. However, limited research has been conducted regarding the cytotoxicity and cytocompatibility of varnishes on oral cells. Recent studies have shed light on the cytotoxic effect of these varnishes on human fibroblast cells.

Material and Methods

The fibroblasts were isolated and cultured in 0.00001, 0.0001, 0.001, 0.01, 0.1, and 1 % fluoride concentration The cells were incubated for 72 hours at a temperature of 37°C and cell viability after the application of varnish was assessed using 3-(4,5-dimethylthiazol-2-yl)-2,5 diphenyl tetrazolium bromide (MTT) assay.

Results

This study observed that fluoride varnish had a concentration dependant cytotoxic effect on human gingival fibroblasts (hGFs). As the concentration of fluoride increased, the cell viability decreased. At 1% concentration, there was maximum cell cytotoxicity. At the lowest concentration (0.00001), more than 78% of the cells were found to be viable.

Conclusion

Further research is necessary to develop safer and more biocompatible fluoride varnish formulations to ensure their efficacy in preventing dental caries without causing harm to oral tissues.

## Introduction

Untreated dental decay is a significant problem in oral health as it can result in pain, tooth loss and infections. There are several measures that can be implemented to prevent dental decay, which include maintaining the hygiene of the mouth, utilizing preventive measures such as sealants, and incorporating fluoride compounds like fluoridated gels and varnishes [1,2]. The Caries Management By Risk Assessment (CAMBRA) protocol, recognized globally, serves an essential part in identifying the underlying causes of dental caries through personalized patient risk assessments [3]. This approach then guides the development of tailored preventive guidelines for each individual, taking into account their specific risk factors. Sodium fluoride treatment is highly recommended for individuals of all ages, with particular emphasis on young patients, those susceptible to dental caries, and white spot lesions in patients requiring orthodontic braces [4]. This preventive strategy assists in strengthening tooth enamel, increasing its resistance to acid assaults and decreasing the incidence of cavities, and in people with decreased salivary flow, especially in the elderly to decrease the incidence of caries [5].

When comparing various fluoride-based preventive treatments, it is observed that varnishes offer longer contact with the tooth surface compared to fluoride gels. Fluoride varnishes are highly effective topical method for delivering concentrated fluoride directly to the teeth. They are applied as a thin layer on the tooth surface, where they adhere and gradually release fluoride over time. This prolonged exposure to fluoride helps in strengthening the tooth enamel and offers sustained protection against dental decay [6]. The most commonly used fluoride varnishes are available in two concentration: 0.1% and 2.26% fluoride. Recent studies have indicated that a 0.1% concentration of fluoride may not provide significant benefits in terms of preventing dental decay. The optimal concentration of fluoride for preventive treatments, such as fluoride varnishes or gels, is typically higher, ranging from 2.26% to 5%. These higher concentrations have been found to be more effective in remineralizing enamel, inhibiting bacterial growth, and reducing the risk of caries development and in preventing caries in elders [7]. It's important to consult with dental professionals to determine the appropriate concentration of fluoride treatment based on individual needs and risk factors when compared to 2.2% fluoride which is the recommended amount in commercially available products. These varnishes are only recommended by the American Dental Association (ADA) in kids below six years of age to lower the danger of accidental ingestion and controlled application when compared to fluoride gels or mouthwashes [8]. Currently, there are approximately 30 available fluoride varnishes with different compositions available, aiming to enhance their antibacterial or remineralizing effect. Antimicrobial agents like chlorhexidine, triclosan, and povidone-iodine are commonly added to mouthwashes, toothpaste, and fluoride varnishes to combat cariogenic bacteria, reduce plaque buildup, and alleviate gum inflammation. Chlorhexidine is a broad-spectrum antimicrobial that inhibits bacterial growth, while triclosan specifically targets plaque-causing bacteria. Povidone-iodine, an antiseptic, offers broad-spectrum antimicrobial activity and helps control the bacterial load. These agents enhance oral hygiene practices and provide additional protection against dental decay, plaque, and gingival issues. It is essential to follow usage instructions provided by dental professionals and product manufacturers for effective and safe utilization [9,10]. 

When fluoride varnish is applied to the tooth surface, the nearby gingival tissue is quite likely to come into direct touch with it. To ensure compatibility with dwelling living tissues, it is important that materials should have no cytotoxic effect [11-13]. There is a growing recognition of the importance of studying the impact of fluoride varnishes on oral mucosa, as certain fluoride varnishes have demonstrated adverse effects on gingival cells and are found to be cytotoxic. This emphases the need for further research in this area. The biocompatibility of numerous dental materials like calcium silicate based endodontic sealers has been thoroughly studied, but there has only been a small amount of research done particularly to observe the cytotoxicity of these materials on oral cells [14,15]. In order to determine whether a substance will have a biological reaction at 0.00001, 0.0001, 0.001, 0.01, 0.1, and 1 %, cytotoxic tests are essential. The cells were incubated for 72 hours at a temperature of 37°C to serve the purpose of assessing potential health risks. This study's objective was to assess the cytocompatability of presently available fluoride varnish and determine its effects on oral fibroblast cells.

## Materials and methods

Extraction and cultivation of human gingival fibroblasts (hGFs)

In adherence to the ethical guidelines established by the University, the cells were isolated and cultured for the study. All participants concerned provided written informed consent. Impacted tooth extraction provided the source of gingival tissues (n = 3). The required tissue was excised and dissolved at 37°C for a time frame of two hours using collagenase I at a ratio of 3 mg/mL (Sigma-Aldrich) and dispase II with a titration of 4 mg/mL (Invitrogen). The obtained cells were placed in a growth medium of Dulbecco's Modified Eagle Medium (DMEM; Gibco, Thermo Fisher Scientific, Carlsbad, United States), which was enriched with fetal bovine serum of 10%. Passaging was performed until the cells reached optimum density and the cells obtained were used in the study.

Preparation of fluoride extract

To achieve 10% concentration, the process involved fusing each topical fluoride coating with DMEM at a ratio of 1:9. This concentration was filtered and the resulting extracts were further adulterated to create different dilutions of 0.00001, 0.0001, 0.001, 0.01, 0.1, and 1 % fluoride concentrations. These were sterilized under ultraviolet (UV) light for two hours.

3-(4,5-dimethylthiazol-2-yl)-2,5 diphenyl tetrazolium bromide (MTT) assay

The effect of a lab-customized fluoride varnish on the vital cells was evaluated using the MTT assay. The cells were placed in a microtiter plate containing 96 cells with 1 × 105 cells per ml density. The groups were subjected to treatment for twenty-four hours. After the treatment period, to each well MTT stock solution was added and placed at 37°C for four hours in an incubator. The crystals were then dissolved and it was measured in a microplate reader (Spectra Max M5, Molecular Devices, USA). The percentage of live cells was measured using the equation: Cell viability (%) = (A570 of (sample)/A570 of (control)) x 100

In addition to the MTT assay, a Live/Dead assay was performed to assess cell viability. 

Fluorescence imaging 

With a confluency of 1x106 cells per well, fibroblast were planted in six-well plates. A eBioscience™ Calcein AM Viability Dye (UltraPure Grade) was applied, incubated for thirty minutes, and then rinsed with 1xPBS (Phosphate Buffer Saline) after the cells had been cultivated for 24 hours. Inverted phase contrast fluorescent microscopy (Invitrogen, evos) was also used to view the cells. Only alive cells that fluoresced green were stained with calcein-acetoxymethyl ester (AM). For each cell state, the number of live and dead-labelled cells was manually counted, and a ratio of live to dead cells was computed. Using the analyse particles measurement in Fiji, cellular aspect ratios were determined from cut-off point Live/Dead pictures.

Evaluation of cell death and degeneration by annexin V-Pe/7-Aminoactinomycin D (AAD) staining

To compare the survival of hGFs after exposure to various concentrations of fluoride, the following procedure was conducted:

Cell Culture

hGFs were cultured either in an incomplete growth medium (control) or in a complete growth medium containing extracts of fluoride varnishes at concentrations of 0.00001, 0.0001, 0.001, 0.01, 0.1, and 1 %. The cells were incubated for 72 hours at a temperature of 37°C.

Viability Assessment

To assess cell viability, Annexin-V-FITC and 7-AAD staining was performed according to the manufacturer's instructions (BD Biosciences). Annexin-V-FITC staining was used to identify early apoptotic cells, while 7-AAD staining was used to detect necrotic cells. The combination of these stains allowed for the distinction of different cell populations: viable cells (unstained), early apoptotic cells (Annexin-V-FITC positive), late apoptotic cells (double positive for Annexin-V-FITC and 7-AAD), and necrotic cells (7-AAD positive). The staining method provided valuable information about the cellular status and helped in evaluating cell viability.

Flow Cytometry Analysis

Stained samples were examined using an LSR Fortessa X-20 flow cytometer (Becton Dickinson, Franklin Lakes, United States) after a one-hour staining period. The stained samples could be examined and measured thanks to the flow cytometer.

Data Analysis

The percentages of viable, early-apoptotic, late-apoptotic, and necrotic cells were estimated based on the flow cytometry results. 

Experimental Replicates

Three independent experiments were conducted, and each experiment included triplicate samples for each fluoride varnish. This design ensured robustness and reliability in the data obtained from the study, as it allowed for assessing the consistency and reproducibility of the results across multiple experimental runs.

By following this procedure, the impact of different fluoride varnishes on hGF viability was evaluated, providing insights into their potential effects on cell health and survival.

## Results

MTT assay

**Figure 1:**
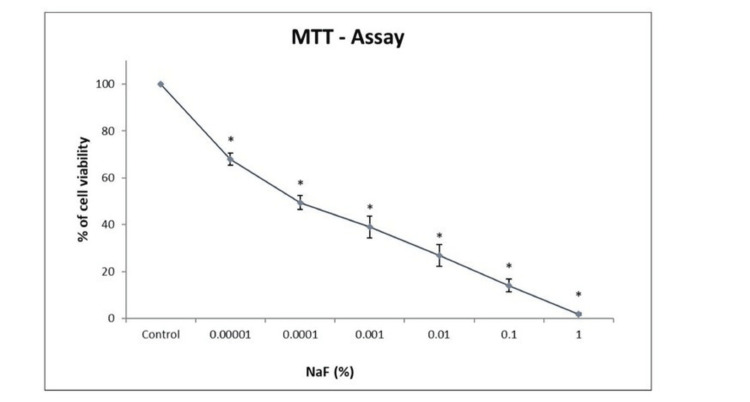
Cytotoxic potential of different concentrations of fluoride varnish (NaF) in primary hGFs treated for 24 hours n = 3 *P < 0.001 vs control MTT: 3-(4,5-dimethylthiazol-2-yl)-2,5 diphenyl tetrazolium bromide; NaF: Sodium fluoride; hGF: Human gingival fibroblast

The effect of the concentration of the fluoride varnish on the hGF activity was analyzed. The fibroblasts were cultured on the growth medium alone (control) or using various concentrations of fluoride (0.00001, 0.0001, 0.001, 0.01, 0.1, and 1 %) for 24 hours. As depicted in Figure 1, as the concentration of fluoride increased, the cell viability decreased. At 1% concentration, there was maximum cell cytotoxicity. 

Apoptosis and necrosis were regulated to assess the toxic effects on cells of various fluoride concentrations on hGFs. This was performed by using annexin-V and 7-AAD to stain it. At the lowest concentration (0.00001), more than 78% of the cells were found to be viable. As the fluoride concentration rose, cell viability decreased.

Immunocytofluoresence staining

**Figure 2:**
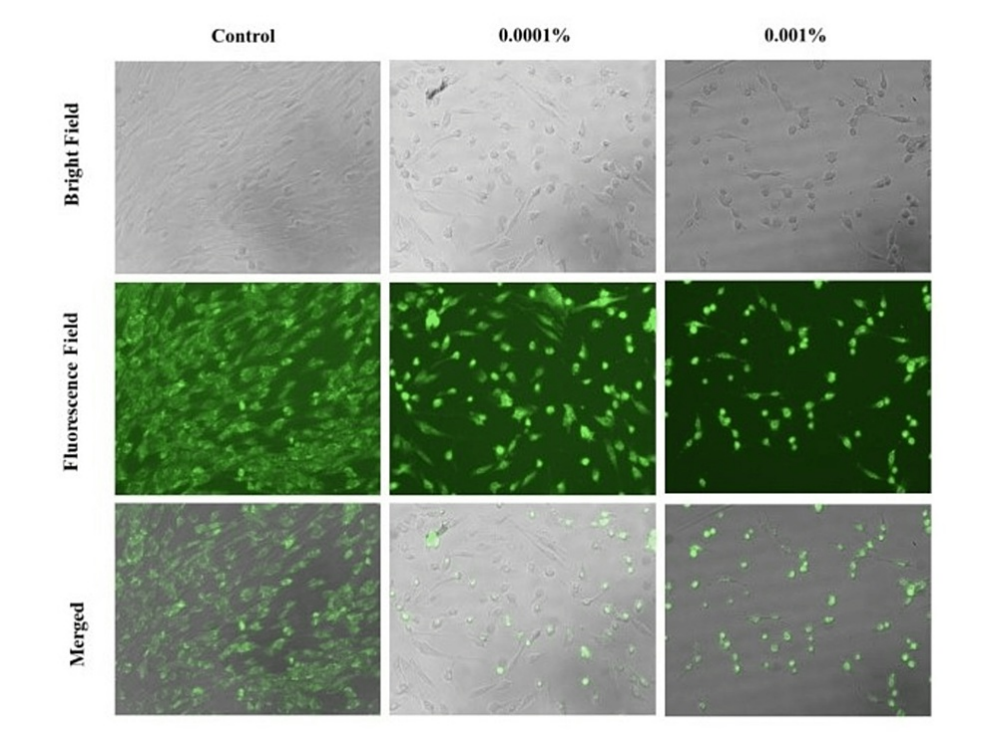
Action of NaF on hGFs under confocal fluorescence microscope Morphology of hGF treated with different concentrations of NaF for 24 hours at 20x. Over the period of incubation self inducing apoptosis of the cells is seen. NaF is seen to be cytotoxic to normal gingival fibroblast cells hGF: Human gingival fibroblast; NaF: Sodium fluoride

To investigate the alterations in cell morphology, confocal fluorescence microscopy was utilized to examine the cells after a 24-hour period. The fibroblasts were observed at control, 0.0001, and 0.001% fluoride concentrations (Figure 2). The fibres at 0.0001% concentration were similar to that of the control group with well-defined cells with a spindle-shaped morphology. At 0.1% concentration, the cells appeared smaller, unattached with some cells exhibiting aberrant morphology. 

 

## Discussion

In the current research conducted, we investigated the biological effects of a commercially available fluoride varnish on hGFs in vitro. Specifically, we examined cell viability, apoptosis, migration, and morphology as indicators of the varnish's impact on hGFs. The results acquired in the current research rejects the null hypothesis, indicating that the application of the varnish had an effect on gingival fibroblasts.

Interestingly, the existing literature lacks extensive research on the cytotoxicity of commercially available fluoride gels and varnishes. Few studies have specifically analyzed the potential negative impact of the components present in fluoride varnishes on oral tissues [13-15].

Our findings contribute to the need to understand the biological effects of fluoride varnishes on hGFs and highlight the need for further research in this area. It is important to continue investigating the cytotoxicity and potential adverse effects of fluoride varnish to ensure their safety and optimal use in dental care, particularly the gingival fibroblasts [15,16]. 

As per the regular instructions, care is to be taken to brush the surface of the teeth with fluoride varnish and avoid any contact with the hGFs. This is usually the reason why dentists finish full dental rehabilitation before applying fluoride varnish on any deep carious lesions. This study used hGF as these are the usual cells most likely to be exposed to varnish in spite of exerting caution. Various studies have been conducted to evaluate dentin hypersensitivity and cytotoxicity of varnish on hGFs or fibroblast cells of a different origin. A study by Verma et al. evaluated the cytotoxicity of a novel varnish [17]. Another study by Lopez et al. tested the cytotoxicity of different types of cement used in dental implants [15].

Biocompatibility is a critical requirement for dental products, ensuring their safety and compatibility with oral tissues. The International Organization for Standardization (ISO) has established standards to guide in vitro biocompatibility studies for dental materials. ISO 7405 and ISO 10993-5 are two important standards that outline the guidelines for evaluating the biocompatibility of dental goods. While various research have been conducted to assess the biocompatibility of various dental materials, it is important to note that each material may have unique characteristics and interactions with oral tissues. Therefore, comprehensive evaluations following the ISO standards are necessary to ensure that dental materials meet the required biocompatibility criteria. By adhering to the ISO standards and conducting rigorous biocompatibility studies, dental manufacturers and researchers can assess the potential risks and benefits associated with dental materials [13-16]. This information is crucial for making informed decisions about the safety and suitability of dental products for clinical use [18].

It is challenging to make meaningful comparisons of the results from different studies due to the utilization of diverse in vitro models, including variations in cell cultures, types of culture medium, compositions and concentrations, as well as different durations of exposure to biomaterials [19]. Consequently, it is imperative to assess the cytotoxicity of different biomaterials using standardized experimental models and appropriate benchmarks. This approach enables the comparison of research findings across studies and ensures a more comprehensive evaluation of the results.

Multiple studies have reported that the ingredients ethyl acetate and cetylpyridinium chloride found in Cervitec F and Fluor Protector S, respectively, exhibit cytotoxic effects on cells and contribute to the development of bacterial resistance. These findings raise concerns about the safety and efficacy of these ingredients. Consequently, there is a growing consensus among researchers that it is necessary to consider alternative ingredients or formulations to replace ethyl acetate and cetylpyridinium chloride in dental products. This shift towards safer and more effective alternatives is crucial to ensure the well-being of patients and to mitigate the potential risks associated with cytotoxicity and bacterial resistance [20].

The application of fluoride compounds has the potential to influence the healing process, either by accelerating or slowing it down. This encompasses the investigation of cellular migration using this technique by previous authors, specifically examining the migration of various oral cells, such as human mesenchymal stem cells from the apical papilla, human periodontal ligament stem cells, and human pulp fibroblasts [21-23].

One limitation of the current study is the absence of trials to evaluate the ions produced by different desensitizers and their potential impact on biocompatibility. This lack of investigation into the specific compositions of the generated ions could be considered a shortcoming of the research. In fact, earlier research reported that low sodium fluoride levels speed up wound healing by promoting cell migration and proliferation.

## Conclusions

This study emphasizes the cytotoxicity and biological effects of a commercially available fluoride varnish on hGF cells. This study has certain limitations which are that it was conducted in laboratory conditions which may vary from clinical scenarios and only one brand of varnish was used in this research. The current commercially available products have high concentrations of fluoride which are cytotoxic in nature. It is necessary to conduct more studies to fully comprehend the processes underlying these effects and to create new fluoride varnish formulations that are safe for oral tissues as well as effective at preventing dental caries. Precautions must be taken by dentists to avoid exposure of varnishes onto the gingival tissues. 
